# Practical considerations for large-scale gut microbiome studies

**DOI:** 10.1093/femsre/fux027

**Published:** 2017-06-30

**Authors:** Doris Vandeputte, Raul Y. Tito, Rianne Vanleeuwen, Gwen Falony, Jeroen Raes

**Affiliations:** 1 KU Leuven, Department of Microbiology and Immunology, Rega Institute, Herestraat 49, B-3000 Leuven, Belgium; 2 VIB, Center for Microbiology, Herestraat 49, B-3000 Leuven, Belgium; 3 Microbiology Unit, Faculty of Sciences and Bioengineering Sciences, Vrije Universiteit Brussel, Pleinlaan 2, B-1050 Brussels, Belgium; 4 Universiteit Antwerpen, Productontwikkeling, Ambtmanstraat 1, B-2000 Antwerpen, Belgium

**Keywords:** gut microbiome, faecal sample preservation, cold chain management, user experience faecal sampling

## Abstract

First insights on the human gut microbiome have been gained from medium-sized, cross-sectional studies. However, given the modest portion of explained variance of currently identified covariates and the small effect size of gut microbiota modulation strategies, upscaling seems essential for further discovery and characterisation of the multiple influencing factors and their relative contribution. In order to guide future research projects and standardisation efforts, we here review currently applied collection and preservation methods for gut microbiome research. We discuss aspects such as sample quality, applicable omics techniques, user experience and time and cost efficiency. In addition, we evaluate the protocols of a large-scale microbiome cohort initiative, the Flemish Gut Flora Project, to give an idea of perspectives, and pitfalls of large-scale faecal sampling studies. Although cryopreservation can be regarded as the gold standard, freezing protocols generally require more resources due to cold chain management. However, here we show that much can be gained from an optimised transport chain and sample aliquoting before freezing. Other protocols can be useful as long as they preserve the microbial signature of a sample such that relevant conclusions can be drawn regarding the research question, and the obtained data are stable and reproducible over time.

## Teaser

The authors review currently applied collection and preservation methods for gut microbiome research, discussing aspects such as sample quality, applicable omics techniques, user experience and time and cost-efficiency, and evaluate the protocols of the Flemish Gut Flora Project, a large-scale gut microbiome sampling effort in Belgium, to give an idea of perspectives, and pitfalls of population-wide studies implementing faecal sampling for gut microbiome research.

## INTRODUCTION

In recent years, the human gut microbiota (the collective microbial content of the intestinal tract) has emerged as a primary target area for health monitoring and modulation (Cho and Blaser 2012; Lozupone *et al.*^2012^). Alterations in the gut microbiota have been linked repeatedly to pathological states such as infections, autoimmune disorders, inflammatory bowel diseases and cancer. Aside from these pathologies, accumulating evidence suggests that gut microbiota composition can serve as indicator of chronic suboptimal health and well-being: either directly linked to suboptimal bowel functioning (e.g. bloating, flatulation, constipation) or extended to general health (Claesson *et al.*^2012^; Koboziev *et al.*^2014^) (e.g. chronic undefined inflammation (Lozupone *et al.*^2012^; Le Chatelier *et al.*^2013^), anxiety and stress (Cryan and Dinan 2012)).

The first major insights on disease-associated microbiome variation have been gained from targeted, medium-sized (N < 400), cross-sectional studies (Koren *et al.*^2013^; Le Chatelier *et al.*^2013^). Many of these early studies collected only limited additional data on the study subjects (e.g. food habits, clinical parameters) and were often single centre-based or restricted to certain populations (e.g. US or Chinese citizens). However, to effectively tackle health monitoring and modulation through the gut microbiota, substantial targeted research efforts are required (Debelius *et al.*^2016^; Falony *et al.*^2016^).

First, larger, representative population cohorts need to be screened in order to pick up relevant microbiome signals beyond multiple expected confounding factors. Many parameters have already been reported to influence gut microbiota composition, ranging from host genotype (Frank *et al.*^2011^), nutrition (Kau *et al.*^2011^), inflammation (Cani *et al.*^2008^) and antibiotic usage (Lozupone *et al.*^2012^) to stool consistency (Vandeputte *et al.*^2016^), but this list is still expanding and the relative importance of these and other unknown factors remains unclear. It has become obvious that upscaling is required to disentangle the multiple, confounding effects in the high-dimensional microbiome data (Raes, Foerstner and Bork 2007; Caporaso *et al.*^2011^).

Second, only a longitudinal study design allows encompassing the dynamic nature of these factors and the identification of microbiome-based prognostic signals and markers. To further study the temporal stability of the gut microbiota, it is also crucial to collect samples over time. The historical lack of sufficiently powered, comprehensively phenotyped, longitudinal studies leads to the baffling observation that it is still unclear what defines a dysbiotic gut microbiota. Recent findings remain conflicting (Finucane *et al.*^2014^), suggesting that there are still unknown confounders blurring research results or, alternatively, that multiple unhealthy microbiome states, in conjunction with host parameters, could be associated to suboptimal health and ultimately disease.

The longitudinal screening of large, representative population cohorts makes it possible to identify microbiome confounding factors together with their relative contribution and to define dysbiotic or suboptimal microbiome states. Given the high interest of the general public for microbiome research, the opportunities to set up citizen-based studies are ample. However, design of such studies implies substantial logistic and financial challenges imposed not only by the required scale but also by the nature of the sampled material. Ideally, faecal material intended for microbiome monitoring needs to be frozen immediately after sampling in order to stop the growth of residing bacteria and potential contaminants and to conserve baseline microbial abundances. Subsequently, samples should be stored at –80°C until DNA extraction (Bahl, Bergström and Licht 2012; Cardona *et al.*^2012^; Santiago *et al.*^2014^). As sampling is often performed in the comfort of the participants’ home, the latter could cause a significant logistical burden. Furthermore, faecal microbiome monitoring efforts risk to suffer from selection biases and drop-out associated to personal aversion towards faecal sampling—especially when sampling procedures are experienced as overly laborious (Sackett 1979; Jordan *et al.*^2013^). The substantial logistical expenses associated to freezing protocols and concerns about ease and user friendliness of faecal sampling has led to a wide range of sampling protocols (both freezing and room temperature(RT)based), all intended to simplify this process. However, given the differential lysis sensitivity between gram-positive and gram-negative faecal bacteria (Wada *et al.*^2012^) and the fact that cell-free DNA can be degraded through oxidation, hydrolysis and enzymatic degradation (Lindahl 1993), sampling collection and preservation methods are thought to have a profound effect on the outcome of sequencing-based technologies used for microbiome determination (Cardona *et al.*^2012^; Gorzelak *et al.*^2015^). Although the variation induced by collection and preservation conditions does not overcome interindividual variation, these kinds of technical sources of variation often do have sizeable effects on the structure of the microbial community when compared to other biological factors (Debelius *et al.*^2016^). Such deviations from the original microbiome composition limit the discovery of relevant biological effects and the possibilities for interstudy comparisons or meta-analyses (Gorzelak *et al.*^2015^; Debelius *et al.*^2016^). Method standardisation would therefore greatly benefit the field: it would facilitate robust biological conclusions and their translation into medicine. Several standardisation efforts to identify sources of lab-to-lab variation and propose standard processing pipelines have already been initiated (Aagaard *et al.*^2013^; Dubilier, Mcfall-ngai and Zhou 2015; The International Human Microbiome Consortium 2015).

In order to guide future faecal microbiome research projects and standardisation efforts, we here review currently applied collection and preservation methods. Despite the importance of downstream processing, we only discuss this first step in microbiome monitoring, as it has been shown to have a profound effect on the microbial community and dictates which omics techniques can be applied (Cardona *et al.*^2012^). We start with an overview of preservation methods and their effect on the observed microbial composition. Next, we elaborate on user experience of faecal sampling and factors affecting drop-out. Lastly, we check time and cost-efficiency of pre-processing pipelines, covering sample collection, transport and aliquoting. This last section includes an overview of faecal sample collection options and transport chains. We illustrate several aspects using our experience in one of the largest microbiome cohort initiatives to date—the Flemish Gut Flora Project (FGFP)—and with this hope to give the reader an overview of options and pitfalls of large-scale faecal sampling for gut microbiota research.

## CURRENT OPTIONS FOR FAECAL SAMPLE PRESERVATION

The first consortia that sequenced the gut microbiota on a (at that point) large scale opted for freezing whole faecal samples as soon as possible at –80°C—either after storage in participants’ home freezers (MetaHIT) (Qin *et al.*^2010^) or in an isolated box with cooled gel packs for a max of 24 h (HMP) (Gevers *et al.*^2012^). Since then a multitude of alternative sampling and storage methods have been developed in order to increase user experience or to allow more flexible transport schemes. Instead of immediate freezing, samples are stored at 4°C or RT for several hours, days or even weeks, with or without stabilisation buffer. Here, we will discuss a selection of some of today's most popular alternatives, each with their respective practical advantages and disadvantages (Table 1). Please note that perhaps the most important aspect—the effect of using these methods on the derived microbiota profiles—is discussed in a separate section.

**Table 1.** Overview of applicable omics techniques, advantages and disadvantages, and a quality assessment of the observed microbiome composition of currently used or tested sample preservation options based on our interpretation of the significant effect of different storage methods from Table 2, with the advised storage period.

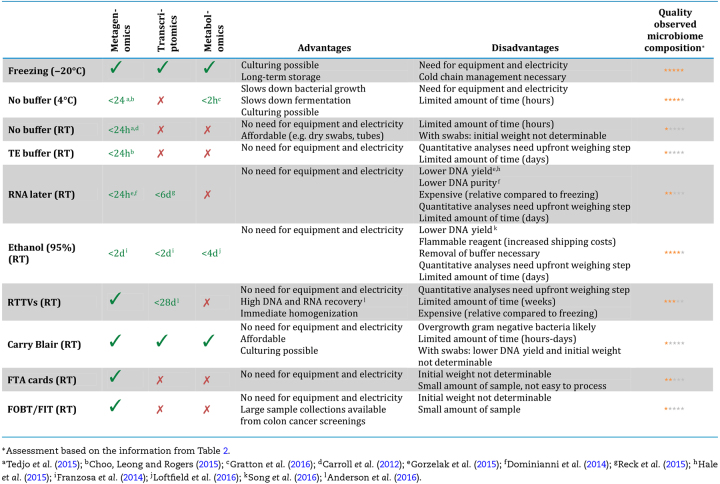

### Keep it cool

When immediate preparation or freezing of a faecal sample is not possible, refrigeration (4°C) can bridge the time between collection and processing. The lower temperature slows down bacterial growth and thus limits deviations from the original composition of the egested stool. Samples processed within 24 h have been shown to be suited for metagenomics analyses (Choo, Leong and Rogers 2015; Tedjo *et al.*^2015^). However, for metatranscriptomic analyses, a stabilisation agent is generally advised in addition to the cooled storage (Reck *et al.*^2015^). As refrigeration also slows down fermentation reactions, it allows subsequent metabolomic analyses with lower differences compared to the original sample. However, storage time should in this case be restricted to 2 h and one has to be aware that volatile components will have been lost (Gratton *et al.*^2016^).

### Stabilise it

The possibility to store samples for several days or weeks at RT allows cheaper logistic solutions and much more flexibility. Hence, several stabilisation buffers have been explored in the hope of reducing the induced variation in microbiota composition. Such solutions however induce lysis of bacterial cells, and consequently these methods are not suitable for combined culture-based approaches, which are rapidly gaining importance for follow-up functional studies.

The commonly usedTris-EDTA-buffer, which is used to solubilise purified DNA or RNA, protecting those from nuclease activity, was one of the first methods investigated (Choo, Leong and Rogers 2015). This buffer contains Tris, a pH buffer, and EDTA, a cation-chelating agent, and is highly effective in preventing nucleic acid degradation. Although faecal samples stored at RT in Tris-EDTA buffer render sufficient high molecular weight DNA to allow 16S rRNA microbiota profiling and shotgun metagenomic sequencing, there is no guarantee that the obtained DNA reflects the original microbial community present in the stool sample (Choo, Leong and Rogers 2015), making its use in metagenomic analyses thus doubtful. Other -omics techniques are generally not applied with this buffer.

Another obvious candidate for microbiome stabilisation purposes is the RNAlater solution. This buffer was originally developed to protect RNA of small tissue or blood samples from degradation by inactivating RNases. However, it is also possible to extract genomic DNA from RNAlater stored samples, thus opening up opportunities for RT storage of faecal samples intended for microbiome research. Samples stored with RNAlater indeed yield sufficient DNA for metagenomic analyses, although both DNA quantity and purity are lower when compared to other storage methods (Dominianni *et al.*^2014^; Gorzelak *et al.*^2015^). As RNAlater is supposed to stabilise RNA, transcriptomic analyses are in theory an option as well. Although high-quality RNA can be obtained from faecal samples stored at RT in RNAlater for up to 6 days, the reagent has been shown to introduce a bias in the mRNA profile, possibly dependent on the abundance of the resident species (Reck *et al.*^2015^). Protein-based techniques are not feasible given the induced protein degradation, nor are metabolomics due to the storage temperature and buffer (Vuckovic 2012). Proper stabilisation of the nucleic acids requires RNAlater to rapidly penetrate the sample, making small sample sizes and large amounts of reagent necessary. In general, a 1:5 ratio sample to RNAlater volume is recommended. With the current pricing of RNAlater (about 1 €/ml), this requirement makes the collection of larger amounts of sample (e.g. for biobanking) cost-prohibitive and complicates the logistics by substantially increasing the required storage space.

Ethanol, widely used in zoology as a medium for tissue conservation, was also investigated early on for its stabilising properties. Optimal DNA preservation is obtained with ethanol concentrations of 95%–99% using a volumetric ratio of 5:1 or higher. Pure ethanol is not recommended as it can contain traces of benzene, seriously affecting DNA integrity, while higher dilutions lead to higher degradation rates (Prendini, Hanner and DeSalle 2002; Nagy 2010). Although ethanol is considerably cheaper than RNAlater (5–10 €/l), it is clear that given the volumetric ratios recommended, it suffers from the same drawbacks regarding storage space. Furthermore, care needs to be taken that the ethanol does not evaporate and as ethanol is not only volatile but also flammable, it is considered hazardous and shipping costs increase accordingly (Nagy 2010; Song *et al.*^2016^). Faecal samples stored under various dilutions of ethanol, with or without a subsequent lyophilisation step, were shown to be suited for genomic DNA as well as steroid hormone analyses (Murphy 1990; Khan *et al.*^2002^; Nsubuga *et al.*^2004^). However, removal of the ethanol prior to DNA extraction is required. This can be achieved using silica beads or drying at a temperature below 60°C for several minutes (Nsubuga *et al.*^2004^; Nagy 2010). DNA/RNA quality of ethanol stored faecal samples is sufficient for metagenomic and metatranscriptomic analyses (Franzosa *et al.*^2014^) as well as other omics assessments (Loftfield *et al.*^2016^). However, culturing procedures are excluded with ethanol-based protocols (Tapani *et al.*^1996^).

Room temperature transport vials (RTTVs) are all in one systems for easy collection and stabilisation of microbial DNA from faeces. The currently available RTTV OMNIgeneGUT requires the participant to transfer a faecal sample of around 500 mg to an opened tube with a spatula. The sample is then forced through a filter by closing the tube with a screw cap. The sample is homogenised immediately in the stabilising buffer by shaking it vigorously for at least 30 s. In this way, participants thus assist in aliquoting and homogenisation, although they do not possess any lab equipment or skills. Consequently, there is a risk that these processes might not completely meet lab standards. Obviously, all participant-dependent methods suffer from this drawback to some extent. According to the manufacturer, storage in OMNIgeneGut tubes maintains the original microbiota composition at RT for up to 60 days and renders high-quality DNA suitable for metagenomic analyses (http://www.dnagenotek.com). DNA and RNA yields are indeed high, and were even reported to exceed those of frozen samples (Anderson *et al.*^2016^). Yet, the reported 74% and 1000% increases in respectively DNA and RNA are probably overestimations, as dilution differences between fresh and frozen samples were not taken into account (Anderson *et al.*^2016^). Current RTTVs are not suited for proteomic, metabolomic or culturing approaches.

### Take it easy

While the methods described above mostly try to reduce deviations from the original microbiota composition (with increased logistic costs), others, such as swab and card-based protocols, are more directed towards facilitating the sampling process for study participants.

Faecal swabs quickly entered the field of gut microbial research because of their user friendliness: participants only have to dip a swab into the collected faeces and put it into a tube. Swabbing can be combined with any of the preservation methods described above, but the tiny amounts of collected sample as well as the storage matrix cause some additional drawbacks (see below).

Most of the commercially available swabbing kits are used for the detection of enteropathogens and use media developed to make culturing of the resident bacteria possible (e.g. Cary-Blair medium) (Tedjo *et al.*^2015^). Although these kits make it possible to use the obtained samples for all omics techniques as well as culturing, overgrowth of gram-negative (Proteo-)bacteria is very likely. Furthermore, the preservation medium dilutes the already small amount of sample, DNA yields are generally low and an adapted DNA isolation protocol is necessary in order to retain sufficient DNA for microbiome research (Tedjo *et al.*^2015^).

Swabs without any preservation medium can be regarded as a special case of RT storage without buffer and consequently contamination or overgrowth of aerobic species cannot be excluded. Because the sample is left to dry, transcriptomic and metabolomic as well as culturing techniques are not possible in this case. Dry swabs were the method of choice for the American Gut Project, one of the first large-scale faecal sampling initiatives.

Nucleic acid stabilising cards, such as the Flinders Technology Associates (FTA) cards, stabilise DNA and RNA by trapping the nucleic acids in the fibres of the card matrix after a lysis reaction. Samples are collected by applying a thin layer of faeces within the indicated region of the card. Although FTA cards have been proven to sufficiently stabilise DNA for a broad range of applications (Smith and Burgoyne 2004; Mas *et al.*^2007^), DNA yields for faecal samples are low compared to other collection methods (Nechvatal *et al.*^2008^).

Multiple colon cancer screenings efforts make use of similar collection methods, namely faecal occult blood test (FOBT) cards or tubes to detect blood in stool, one of the early-onset markers of colon cancer. The sampling methods applied are of particular interest, as they would open up additional large-scale epidemiological research possibilities. Colon cancer screenings usually apply either the guaiac-based FOBT (gFOBT) or faecal immunochemical Test (FIT), although high-sensitivity versions of the latter are nowadays recommended (Leddin *et al.*^2010^). While there are several commercially available gFOBTs and FITs, most adhere to a collection procedure in which three consecutive samples are taken using a swab—either smeared onto a paper card and allowed to dry or stored in a tube with haemoglobin-stabilising buffer—and returned at RT within 2 weeks, usually by mail. However, some protocols include additional steps, such as dipping the swab into toilet water or storage in the fridge. In order to detect blood in the samples both gFOBTs and FITs require the application of reagents. The gFOBT, which is based on the pseudo-peroxidase capacity of heme and its chemical reaction with guaiac, requires a drop of a hydrogen peroxide containing solution to initiate a colour conversion onto the paper, while FIT tests mix part of the obtained sample solution with the antihuman haemoglobin antibody reagent. Either the leftover of the buffer of the FIT or the developed cards could potentially be used for microbiome analyses. However, on top of the high variability in storage conditions, the use of study samples from colon cancer screening projects has some drawbacks worth considering. First, the development solution of the gFOBT card and the stabilisation buffer of the FIT tube might cause additional diverging effects on the observed microbiota composition. Second, the required modifications in diet (e.g. abstinence from red meat) and medication (e.g. avoidance of NSAIDs) for the gFOBT test might influence the results.

The applicability of both card and swab-based methods in microbiome research is further limited by the small amount of material collected. Given the heterogeneity of the stool matrix and the increased effect of contamination, the latter magnifies sample processing errors. Moreover, small sample volumes impose the development of adjusted DNA extraction protocols (Tedjo *et al.*^2015^), making results incomparable with all other methods that do allow standard extraction techniques (Aagaard *et al.*^2013^). Even with proper stabilisation, the use of other omics approaches would be restricted by the tiny amount of collected material. Another drawback of these methods is the difficulty to determine the weight of (part of) the sample. Assessment of the initial weight is invaluable for all quantitative techniques (e.g. metabolomics). The storage matrix and the tiny amounts of sample collected using swab and card-based methods however make determination of the initial weight impossible.

## EFFECT OF PRESERVATION ON OBSERVED MICROBIOTA COMPOSITION

Compared to the amount of ‘applied’ microbiome research papers, only a small number of technical studies have been published regarding the effect of different sampling and storage methods on microbial compositional profiles. The majority of these studies are based on small-scale sampling of healthy adults and might therefore miss or underestimate some effects, limiting the generalisation of their results. For example, less stable or dysbiotic microbiota constellations, such as those of infants or individuals with intestinal disease, might be affected differently by storage conditions (Carroll *et al.*^2012^; Guo *et al.*^2016^). Although limited by their restricted population and sample sizes, these studies do provide important insights into the divergent effects of temperature and stabilisation media over time.

### Cryopreservation

The current gold standard for long-term storage of faecal samples—frozen at –80°C storage without buffer—comes from years of experience in tissue and culture preservation. It is supported by observations that microbiota composition, evaluated by 16S RNA gene sequencing, does not change significantly over 4 weeks (Bai *et al.*^2012^) or 6 months (Carroll *et al.*^2012^) of storage at –80°C compared to samples extracted within 30 min after egestion. Although Bahl *et al.* (2012) reported an increased Firmicutes/Bacteroidetes ratio in seven out of nine qPCR-assessed samples, and Fouhy *et al.* (2015) and Flores *et al.* (2015) observed a similar (though not significant) trend in short-term frozen samples, other studies with more samples and longer storage periods do not confirm these findings (Fouhy *et al.*^2015^; Anderson *et al.*^2016^; Shaw *et al.*^2016^). The effect of long-term freezing on the faecal microbiota has been investigated by Shaw *et al.* (> 2 years) and Kia *et al.* (> 14 years). Despite being sheared more extensively than fresh material, high-quality DNA suitable for 16S rRNA analyses could be recovered from these samples (Kia *et al.*^2016^). Long-term freezing seems to induce few significant changes in microbial community, with a small reduction in the number of observed OTUs and shifts in some specific abundances (notably increased *Lactobacillus* and *Staphylococcus*), but similar alpha- and beta-diversity measurements, confirming stability of the faecal microbiota during long periods of freezing (Shaw *et al.*^2016^).

Cryopreservation media are of standard use in axenic culture collections due to their ability to reduce cellular damage, improving viability and activity recovery. Yet, only few studies have been carried out to study their use in complex communities (Laurin *et al.*^2006^; Vlaeminck *et al.*^2010^; Hamilton *et al.*^2012^; McKain *et al.*^2013^; Waite, Deines and Taylor 2013; Kerckhof *et al.*^2014^), leaving their effect on the observed microbiota composition almost unassessed (McKain *et al.*^2013^; Waite, Deines and Taylor 2013; Kerckhof *et al.*^2014^). Addition of the broadly used cryoprotective agent DMSO did not markedly improve activity recovery (as measured by SCFA production) in a study using faecal biomass, nor did it lead to any significant differences in community composition compared to regular freezing (Kerckhof *et al.*^2014^). Although culturing will be the primary goal of studies applying cryoprotective agents, the collected samples can in theory also be used for omics technologies, making it worthwhile to assess their effect on community composition.

Whether or not cryoprotection agents are applied, any thawing of samples should be avoided for downstream omics analyses as it increases the chance of contamination and microbial blooms, reduces DNA and RNA integrity due to nuclease activity (Cardona *et al.*^2012^; Santiago *et al.*^2014^), and limits metabolomic analyses because of the loss of volatile compounds (Kerckhof *et al.*^2014^) and the continuation of fermentation reactions (Gratton *et al.*^2016^). Freeze–thaw cycles have been shown to induce changes in taxa abundance (Gorzelak *et al.*^2015^; Song *et al.*^2016^), although limited (up to 4) and short (up to 10 min) cycles are reported to have more robust profiles (Gorzelak *et al.*^2015^). Longer thawing periods however make subsequent metagenomic and transcriptomic analyses less trustworthy as this severely reduces DNA and RNA quality: after 1 h of thawing, about 20% of the DNA is fragmented into pieces smaller than 1.5 kb, and RNA integrity numbers drop below 7, the threshold acceptable for conducting metatranscriptomic studies (Cardona *et al.*^2012^).

Other important points to consider when using cryopreservation protocols are the time and storage conditions until freezing as well as the freezing process itself. Short sampling times and fast freezing processes, as applied in flash freezing with liquid nitrogen, are ideal as all biological processes are put on hold very quickly (Fouhy *et al.*^2015^). Especially in the light of transcriptomic and proteomic analyses, instant freezing provides higher quality samples that are more likely to reflect the original state of the sample (Flores *et al.*^2012^; Fouhy *et al.*^2015^). In addition, culturing techniques would benefit of flash freezing as cells suffer less damage than when frozen more slowly (Fouhy *et al.*^2015^). If cryopreservation cannot be initiated immediately, overgrowth of aerotolerant bacteria can be avoided by storing samples under anaerobic conditions, e.g. using gas paks (Wehrspann 1976). Such approaches would also benefit culturing applications, as they increase the viability of anaerobic bacteria.

### RT protocols

The effect of alternative protocols on microbiota composition compared to freezing (Table 2) has been assessed in several 16S RNA gene-based studies. All of these report individual variation to be larger than variation induced by the storage conditions investigated. While RT storage without stabilisation buffer significantly changes taxa abundances from 30 min onwards (Gorzelak *et al.*^2015^; Sinha *et al.*^2015^; Guo *et al.*^2016^; Shaw *et al.*^2016^), refrigeration shows relatively unchanged microbial community profiles when limited to 24 h (Choo, Leong and Rogers 2015; Tedjo *et al.*^2015^). From these reports, it is also clear that the use of preservation buffers to overcome the detrimental effects of RT storage is not always effective. Tris-EDTA and RNAlater stored samples differ in taxa abundances and have altered alpha-diversity measurements (conflicting results) after 72 h (Dominianni *et al.*^2014^; Choo, Leong and Rogers 2015; Sinha *et al.*^2015^; Blekhman *et al.*^2016^). The addition of antibiotics such as kanamycin or ciproflaxin, which prevent RNA transcription or protein translation, does not have any additional stabilising effects when added to RNAlater (Flores *et al.*^2015^). Longer periods of RT storage with RNAlater should definitely be avoided, as stability is seriously impaired after 2 weeks (Song *et al.*^2016^).

**Table 2.** Overview of reported significant effects of different storage conditions on alpha diversity measurements (richness, evenness, Shannon Diversity index (SDI)), beta diversity measurements (e.g. unifrac, Bray–Curtis dissimilarity (BC)) and taxa abundances in comparison to immediate freezing (at –20°C or –80°C).

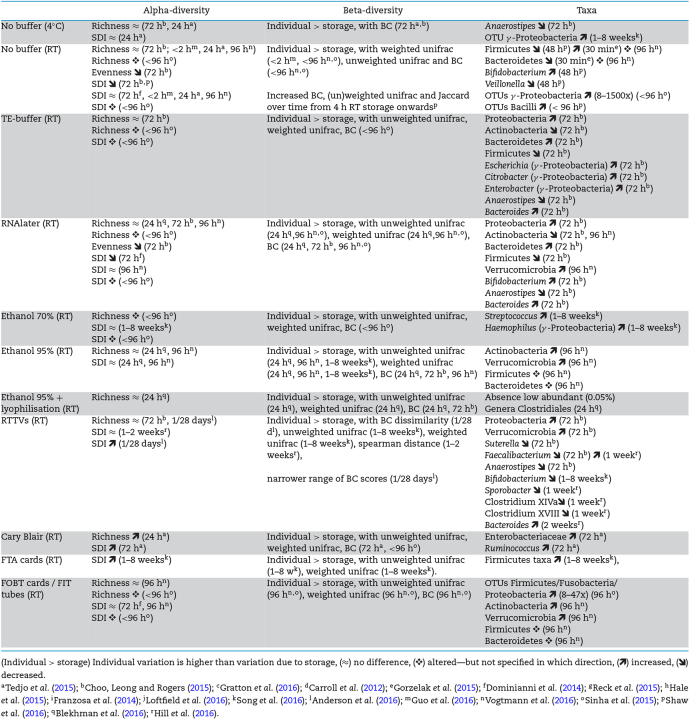

Ethanol does not improve stability at 70% (Choo, Leong and Rogers 2015; Sinha *et al.*^2015^; Song *et al.*^2016^) but seems to have beneficial effects at 95%, providing similar alpha-diversity measurements after 24 h and only mild effects on community composition (Choo, Leong and Rogers 2015; Blekhman *et al.*^2016^; Song *et al.*^2016^).

The RTTV OmniGeneGut is able to preserve faecal material over a longer period of time, but suffers from consistent inadequate detection of *Faecalibacterium* and *Bifidobacterium* (Choo, Leong and Rogers 2015; Hill *et al.*^2016^). According to Anderson *et al.* (2016), inter-replicate variation is less for OmniGeneGut samples, which could be a consequence of the homogenisation. Based on an evaluation of infant and elderly samples with clear differences in richness, Hill *et al.* (2016) hypothesised that the OmniGeneGut vials could have a larger influence on samples with a lower diversity. The effect of storage for more than 60 days, either at RT or frozen, has not yet been established, leaving unanswered questions regarding sample stability for those studies demanding long-term storage.

The process of swabbing, using Cary-Blair medium and conditions simulating mail transport, was recently investigated, and the results suggest that the method might induces most anomalies: swab samples deviated most from their original subject-specific cluster, compared to all other investigated methods, including 24 h RT storage. Swab samples also showed increased alpha-diversity and higher relative abundance of the taxa *Ruminococcus* and Enterobacteriaceae, while *Methanobrevibacter*—despite its presence—was not picked up in any of the samples, due to the dilution by the preservation medium, as suggested by Tedjo *et al.* (2015). Swabs without preservation medium also have profound effects on microbiota composition, with altered abundances up to phylum level and changed alpha-diversity measurements, severely impairing reproducibility (Sinha *et al.*^2015^). The large differences in reproducibility and OTU profiles between two labs performing similar DNA extraction protocols as reported by Sinha *et al.* (2016) might also be partially caused by a swabbing step applied by one of them.

FTA and FOBT tests make use of a swab sample smeared on a card and allowed to dry (FTA and gFOBT) or stored in haemoglobin stabilisation buffer (FIT). The swabbing procedure combined with the prolonged exposure to fluctuating temperatures and oxygen likely induces similar or more pronounced effects on microbiota composition as the earlier reported faecal swabs. Song *et al.* compared samples stored with FTA cards to fresh faecal samples and reported highly correlated OTU abundance profiles over a range of storage conditions. Changes in taxa abundance were not specified but seemed to be consistent, as they could be corrected for using a detrending method based on the mean fold change between abundances in fresh and FTA stored samples. FTA cards tend to recover a greater diversity of bacterial taxa than other preservation methods, and the authors suggest that this might be due to improved lyses of spore-forming Firmicutes through the FTA card matrix. Yet, contamination of the low-biomass sample seems a more likely explanation.

The effect of FOBT tests on the microbial composition was recently explored by Sinha *et al.* (2015) and Vogtmann *et al.* (2016), although under less harsh conditions as those expected to occur during cancer screening. Both FIT and gFOBT bacterial profiles are reported to differ significantly from the gold standard regarding technical reproducibility and stability (Vogtmann *et al.*^2016^). Altered taxa abundances up to phylum level were observed with more than 10-fold change in several OTUs (Sinha *et al.*^2015^; Vogtmann *et al.*^2016^). Development of similarly stored FOBT cards with Hemasensa developer, used to detect blood in the stool sample during colon cancer screenings, induces little variation, as OTU abundances highly correlated between pre- and post-development samples (Sinha *et al.*^2015^). Based on intraclass correlation analyses of general parameters, such as phylum abundance, alpha diversity and the first principal coordinate of a beta diversity metric, the authors, however, rather surprisingly, conclude that FOBT cards and FIT tubes are ‘acceptable for future faecal sample collection in microbiome studies’ (Sinha *et al.*^2015^; Vogtmann *et al.*^2016^).

### What makes a preservation method acceptable?

While some of the methods discussed in the previous section clearly show discrepancies from the original microbiota composition up to phylum level, they were still described as ‘acceptable for future microbiome studies’ by the reporting authors. This of course raises the question as to what makes a method ‘acceptable’. We will therefore discuss some of the essential features and desirable aspects of storage methods for faecal samples intended for microbiome research and assess how the methods discussed relate to these criteria. First and foremost, the microbial signature of a sample needs to be preserved to such extent that relevant conclusions can be drawn regarding the research question. Second, key measures of gut microbiota composition must be stable and reproducible over time. Only after due consideration of the points above, factors such as price, user friendliness and ease of processing should be brought to the table.

The most important criterion when selecting a preservation method is the need to be able to draw relevant conclusions regarding the research question. Next to the accuracy of the obtained data, this criterion obviously relates to the—expected—effect size of the investigated and confounding parameters on gut microbiome variation. Debelius *et al.* (2016) recently sorted some already investigated parameters according to their contribution to gut microbiota composition in animals and humans and identified host species, age and lifestyle as parameters with largest effect size, while antibiotic use was classified as medium, and genetics, xenobiotics, exercise and long-term dietary patterns were considered to have small-to-medium effects (Debelius *et al.*^2016^). Using the FGFP data, we were able to identify the major covariates of gut microbiota composition in healthy individuals and estimated their non-redundant effect sizes between 0.5% and 5% (Falony *et al.*^2016^). The identification of covariates of microbiota composition is the first step in the search for a causal link between gut microbiota and disease or suboptimal health. However, it is becoming increasingly clear that the individual contribution of such covariates is very small. This urges microbiome researchers to be particularly careful in data acquisition. Although the statement that preservation conditions do not overcome differences between individuals is often made as a sort of quality label in research articles comparing different storage methods, procedures might easily mask the biological influences investigated under the conditions applied. Indeed, interindividual variation in microbiota composition is high, generally accounting for 50% to 80% of total variation and overruling all other explored parameters. Such assessments therefore provide no idea whatsoever about the applicability of a certain storage or transport method. What is needed is an estimation of the effect size of the investigated storage condition in microbiome variation. However, none of the articles discussed in the previous sections report this measure. It is therefore impossible to know how much influence a preservation method has on the gut microbiota composition or to compare its effect with that of previously identified covariates. Consequently, it is very difficult to assess whether or not a preservation method would allow to draw relevant conclusions regarding the research question. In addition, one can only make guesses regarding the possibility to conduct meta-analyses on cohorts with differently stored samples. Inclusion of effect size estimations of microbiome variation in future research articles investigating storage influences would therefore certainly advance the field.

Depending on the estimated effect size of the investigated parameter and the sample size, a certain degree of storage-induced deviation from the original microbiota composition can be allowed. It is however important to keep in mind the specific deviations induced by the considered protocols. Indeed, a procedure generating random noise is a whole lot different from one inducing specific alterations in taxa abundances or diversity estimates. An altered detection of key species might for example lead to a bias in disease research. The increased abundance of Proteobacteria reported in samples stored with Tris-EDTA buffer (Choo, Leong and Rogers 2015), OmniGeneGut RTTVs (Choo, Leong and Rogers 2015), faecal swabs (Tedjo *et al.*^2015^) or FOBT cards (Sinha *et al.*^2015^)—which are thought to be a result of the aerobic storage conditions—is particularly worrisome given the resemblance of these changes with some reports of diet-induced variation or disease-associated dysbiosis (Frank *et al.*^2011^; Mondot *et al.*^2011^; Wu *et al.*^2011^; Le Chatelier *et al.*^2013^). *Faecalibacterium, Bifidobacterium* and Verrucomicrobia (comprising *Akkermansia*) are other important taxa related to health status that are regularly reported to differ from the gold standard. Especially in those cases where first assumptions about signature species can be derived from previous studies, the choice of a suited preservation method should be addressed accordingly. In this regard, we want to point out the importance of precise and clear reporting of method comparison studies. As they form a guideline for future work, researchers conducting such studies have the responsibility to report the observed effects regarding genera abundance in a clear and transparent manner. Only reporting phylum level variation or limiting description to those bacteria with a more than 10-fold change in abundance instead of assessing the significance of differences between groups is inadequate, as biological relevant signals can be situated at genus or even species level and are often far <100%. In order to correct abundance tables for the overgrowth of aerotolerant bacteria, mostly Gammaproteobacteria, computational approaches have been suggested (Song *et al.*^2016^). However, assessing the accuracy of such correction methods is difficult and consequently the obtained data are highly uncertain, making such practices of little use for studies aiming to characterise the impact of low-effect-size factors on the gut microbiota.

Despite the lack of information regarding the explained variance of preservation methods on gut microbiome variation, some general recommendations to fulfil the criterion described above can be made. Obviously, the effect of preservation is containable when a single method is applied throughout a study. Many authors report significant differences between the investigated storage methods (Sinha *et al.*^2015^; Song *et al.*^2016^; Vogtmann *et al.*^2016^), and therefore the application of multiple storage methods within a study is unadvisable. In order to control the diverging effect of collection and storage further, protocols should be specified in detail and followed as strictly as possible. These and all measures reducing technical variation due to preservation are a good way to diminish the effect of storage conditions on the observed microbiota composition, whatever the initial accuracy level of the procedure.

A second important point regards the stability and reproducibility of key measures of gut microbiota composition over time. Whatever the effect size of the considered parameter on gut microbiome variation, major markers of gut microbiome research (including diversity indexes, community types or phylum abundance) should not differ significantly between technical replicates. Given the relative stability of such markers within an individual (Wu *et al.*^2011^; Yatsunenko *et al.*^2012^; Donaldson, Lee and Mazmanian 2015), not fulfilling this condition would most probably lead to invaluable conclusions. Most of the methods described meet this minimal requirement (Dominianni *et al.*^2014^; Flores *et al.*^2015^; Sinha *et al.*^2015^; Anderson *et al.*^2016^; Hill *et al.*^2016^; Song *et al.*^2016^; Vogtmann *et al.*^2016^), although swab and card-based techniques might form an exception, especially with prolonged storage times (Sinha *et al.*^2015^; Vogtmann *et al.*^2016^). Of note, technical reproducibility of most methods applying RT storage diminishes with the time of preservation (Sinha *et al.*^2015^; Tedjo *et al.*^2015^; Anderson *et al.*^2016^; Hill *et al.*^2016^; Song *et al.*^2016^).

Only when the microbial signature of a sample can be preserved to such extent that relevant conclusions can be drawn regarding the research question and the obtained data have been shown stable and reproducible over time, other aspects of storage methods can be taken into account. Definitely worth considering are the applicability of standard protocols, which opens up perspectives for meta-analyses, and the possibility for biobanking, which would reduce the cost of follow-up research.

## USER EXPERIENCE OF FAECAL SAMPLING AND FACTORS AFFECTING DROP-OUT

Keeping participants motivated throughout a study is a demanding task in every research project, but is believed to be even more critical in colon microbial research given the possible reluctance for faecal sampling. Despite the long track record of faecal sampling for gastrointestinal research, there is almost no information available regarding sampling aversion or protocol preference and how this affects protocol adherence. Without such information, there is no use in discussing different protocols for microbiome research on user experience, nor can any conclusions be drawn regarding the importance of this aspect and the weight it should get when selecting a suitable sampling method.

To gain more insight on the user experience of the faecal sampling process, we set up an evaluation of the sampling procedures of the FGFP. In order to collect high-quality samples for microbiome research, the FGFP asked participants to immediately freeze their sample into their home freezer and established a permanent collection network of 60 pharmacies across the densely populated region of Flanders, ensuring a collection point within 10 km driving distance of each participant's home address. A first large-scale collection round used this network together with frequent pharmacy-to-laboratory transport on dry ice to collect all faecal samples delivered during a 12-week period. In total, 3083 individuals, of whom one third can be regarded healthy, completed all sampling procedures.

### Why do people drop-out?

During the first FGFP sampling round, 4837 sampling kits were sent to project participants—64% of the addressed volunteers completed the FGFP sampling protocol. To investigate reasons for (lack of) compliance to the FGFP sampling procedure, a link to an evaluation questionnaire was mailed to 500 subscribed volunteers, randomly selected out of all people who received a sampling kit during the first sample collection round and independent of their final decision to participate. Among the 267 responders, 21 indicated not having delivered the samples required. Before registering as FGFP volunteers, potential project participants were informed of the necessity of faecal sampling. Because of the applied awareness policy, none of the responders reported that their decision to renounce from further participation was related to the actual sampling procedure, and only 5% of those completing the full protocol indicated having thought about quitting because of sampling aversion. Most cited reasons for renouncing to participation were the burden of scheduling a GP consultation (20%) and the impossibility of completing procedures within the sampling round timeframe proposed (33%). Although people are not informed upfront that project participation required the storage of faecal samples into their home freezer, only 2.8% of respondents indicated it to be a reason for quitting the project.

### How big is the ‘yuck-factor’?

**Figure 1.**
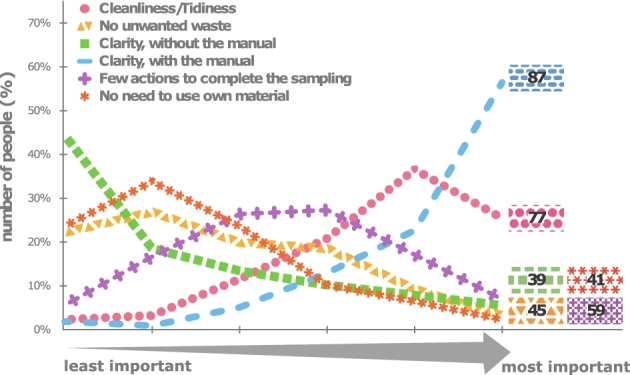
Importance of different features of a faecal sampling procedure. Volunteers were asked to rank six different aspects of the sampling procedure from most important to least important. The figure represents the percentage of people that ranked the feature (y-axis) at a certain level of importance (x-axis). The total score of each feature is given next to its distribution.

When asked for sampling preferences, 56% of effective participants indicated they would opt for a protocol allowing to complete procedures in proximity of the toilet. Most important aspects of faecal sampling procedures were indicated to be (i) the instructiveness of the sampling manual, (ii) sampling hygiene and (iii) straightforwardness of sample handling (Fig. 1). Surprisingly, FGFP participants value a clear description of the required sample handlings over all other factors that could increase user experience. A higher certainty that one performed the sampling correctly, which—based on the reactions in the opinion box—seems to be important for most participants, might explain this result. As FGFP participation is voluntarily, this feeling might be less present in other studies. Nevertheless, it is clear that personal aversion from faecal sampling is probably less important than commonly assumed.

In the case of the FGFP, people were instructed by the sampling manual to collect their sample using a self-provided basket covered with the biodegradable, flushable plastic sheet included in the sampling package. On completion of the collection of their stool sample, they were asked to fill three small plastic transparent tubes with about 1 cm of stool using three provided plastic spoons. They could cut off the plastic spoons with a scissor (not provided) and leave the rest in the tube, so that they would not be left with stool-covered waste. The rest of their stool could be flushed through the toilet using the biodegradable plastic.

Instructiveness of the sampling manual included in FGFP kits was scored (almost) completely clear by 93% of participants. All participants indicated having read sampling instructions before (69%) or during (31%) procedures. While about two-thirds of volunteers judged the sampling procedure as ‘very to reasonably hygienic’, 15% scored it at the low end of the hygiene scale. Suggestions to improve sampling hygiene concerned the use of wider collection tubes (present diameter: 1 cm) and more rigid sampling spoons. Overall, FGFP sampling procedures were judged uncomplicated and about 90% of the participants did not object to the use of own materials (such as a collection recipient and scissors). Most cited inconvenience (30%) was the difficulty to comply with the FGFP requirement to deliver a urine-free sample, which, for obvious anatomical reasons, appears more problematic for women (36%) than men (19%). About 24% of volunteers reporting this issue were not able to take a urine-free sample at the first attempt. As the sampling manual clearly stated the importance of delivering a urine/toiletwater-free sample, 10 out of 13 participants repeated the sampling procedure. However, the latter indicates that up to 1.3% of samples collected are urine-contaminated—with currently unassessed impact on the faecal microbiome.

The faecal sampling procedure as implemented by the FGFP was evaluated positively by most participants. Two-thirds of the volunteers who registered for FGFP participation effectively completed all procedures. Microbiome monitoring efforts using a similar sample collection protocol should hence take into account drop-out ratios of about 40%. In part due to an active awareness policy—participants can consult the sampling manual on the FGFP website prior to registration—no volunteers renounced from effective participation based on the actual sampling handlings. As <5% of people indicated thoughts of quitting related to the faecal sampling procedure or the requirement of sample storage in the home freezer, we can assume the drop-out ratio of microbiome research studies is not heavily influenced by faecal sampling user experience. In this regard, extending the collection network or organising multiple collection rounds—reportedly the main hurdles to complete the sampling process—would probably be more effective ways to decrease this ratio in similar set-up projects. Nevertheless, participants do appreciate a simple and hygienic sampling procedure. Although improvements on this front would without doubt improve user experience, we discovered that even more could be gained from a clear sampling manual. The data of the FGFP evaluation thus enable us to deduce some general recommendations, yet our conclusions are obviously based on a single study and might not be applicable in different circumstances.

## COST AND TIME EFFICIENCY OF PRE-PROCESSING

To scale up microbiota research projects to the extent and duration required, automation of sample handling, DNA extraction and sequencing preparation seem essential in order to keep microbiome monitoring efforts both time and cost-effective. However, resource management might benefit even more from the optimisation of the pre-processing pipeline. We will therefore discuss budget and time investment of several logistic options for faecal sampling.

Before starting DNA/RNA extraction or any other experimental work for microbiome research, a standard protocol for faecal sampling for microbiome research can be divided into three main steps: collection, transport and aliquoting of the sample. Because only a small part of the sample is needed for subsequent protocols, aliquoting is an important step within the pre-processing. Off course, these steps are all influenced by the choice of preservation method. Opting for a specific method for one aspect limits the available options for the others. Because collection and aliquoting are very intertwined, we will first discuss cost- and time efficiency of different combinations of these two steps and then take a closer look at transport logistics. In order to illustrate the impact of the different options on large-scale faecal sampling, we will use a study collecting 10 000 samples as an example.

### Collection and aliquoting

While aliquoting is very straightforward for most RT methods, it can be quite complicated for freezing protocols. Either cutting or drilling a frozen faecal sample is a very laborious, expensive and time-consuming process. However, new devices implementing the principle of aliquoting before freezing—right after collection—were developed recently (e.g. DiviMat (our lab)). Therefore, we can now distinguish three main aliquoting options: (i) aliquoting after RT storage, (ii) aliquoting after freezing, (iii) aliquoting before freezing (Table 3).

**Table 3.** Comparison of the different aliquoting methods for a study collecting 10 000 samples.

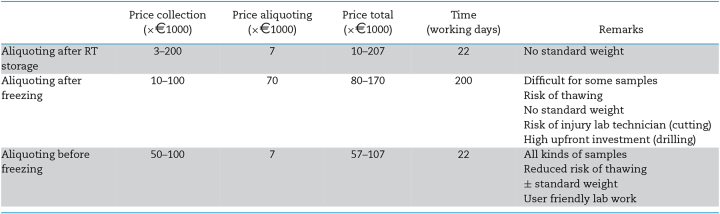

Based on our own experience with the discussed methods, we estimate the time spend on aliquoting to increase with a factor 9 when it needs to be done after freezing of the sample. In case of our example study, methods implementing RT aliquoting—either before freezing or after RT storage—would require only 22 working days, while methods requiring aliquoting after freezing would easily take more than 200 working days. Not surprisingly, for the latter the cost associated to downstream processing is largely determined by the cost of labour, and can go up to 70%, depending on the collection method. In contrast, total budget for protocols able to aliquot at RT highly depends on the price of the collection method, as this can range from €0.3 to €20 (Box 1). The most time-efficient methods are thus without doubt those that perform the aliquoting at RT, but cost-efficiency depends largely on the collection device used.

### Transport

For transport logistics, we distinguish five main transport plans: (i) immediate sampling on location, (ii) home sampling without freezing and regular mail or courier delivery (e.g. American Gut), (iii) home sampling with freezing with a courier transporting the samples from the participant's place to the lab (e.g. Life Lines Deep (Tigchelaar *et al.*^2015^)), (iv) home sampling with freezing using one moving collection point where participants bring their samples, (v) home sampling with freezing using many collection points together with a courier service (e.g. FGFP).

In densely populated areas with an easily accessible laboratory, participants could come to the lab and sample on location. While it is difficult to estimate the compensation required for such an effort, it is clear that the transportation costs of the participant can be used as a guideline. Although highly depending on the situation, we will therefore take into account a reimbursement cost of €15 per sample for our example study, bringing the total budget for transport in this case on €150 000.

The second option, regular or express mailing, is currently the cheapest and quickest way of transporting faecal samples. Protocols making use of swabs, FTA cards RTTV or tubes with non-hazardous buffers stored at RT would be able to do so and spend about €2.5 per sample to receive it within three working days. Transport of 10 000 samples would therefore cost about €25 000. While the usage of regular mailing companies has the advantage of being inexpensive and easy implementable across large geographic distances, it has the disadvantage of an uncontrolled transport chain where temperature profiles depend on transport conditions, which can vary substantially across regions and seasons. Consequently, samples might be subjected to high temperatures or freeze (and thaw) during transport.

All other transport plans discussed here make use of a freezing step and thus require adequate cold-chain management. Based on an evaluation of FGFP procedures, we make suggestions on how to achieve this (Box 2).

Box 1. **Current options for faecal sample collection.**

**Table:** 

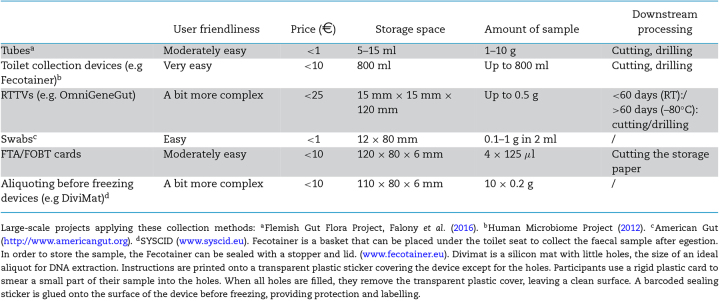

Box 2 **Cold-chain management**

Based on an evaluation questionnaire, we estimate that 97% of collected FGFP samples are frozen within 5 min after sampling. On average, samples are stored in a home freezer for 2 days and 6 h before transport to a collection point. Around 85% of participants indicated an estimated transport time below 30 min. In order to maintain samples in frozen condition during transport—as explicitly stated in the sampling manual—participants report to use a cooling box with cooling elements (67%), ice cubes (6%), an isolating bag/box (18%) or a cooling box (6%). Although 5% of the volunteers indicated not to have cooled nor isolated the samples, this does not necessarily imply thawing, given the short transport times. In addition to the questionnaire, longitudinal temperature profiles of 137 complete sampling-storage-transport processes were collected. On average, the whole sampling procedure takes 8 days and 16 h. A total of 13 out of 137 samples (9%) got defrosted once. Most thawing events occurred during transport from the volunteer's home to the collection point; however, one appeared to be due to an inadequate defrosting cycle of the home freezer. Thawing time of these samples varied between 5 and 50 min. So although most FGFP participants demonstrate their inventiveness to maintain samples in frozen conditions, around 23% opt for minor isolation, and transport from the home freezer to FGFP collection points seems a crucial step, with about 10% of samples at risk of thawing. As 23% opt for minor isolation, providing a cooling cover, which participants can wrap around their samples upon freezing, would most probably decrease this number drastically. As most thawing events occur during transport of the sample, a decreased average distance to the collection point could not only be a remedy for volunteer drop-out, but also a way to limit thawing risk. In order to check on thawing events, a logging device could be added to the sampling package. Currently, there are user-friendly thawing-sensitive strips that indicate a thawing event by colour change available for about €1. Simple visual inspection at the moment of arrival by the lab technician is then enough to identify thawed samples.

A transport plan using home sampling with freezing with a courier transporting the samples from the participant's place to the lab has been applied by Life Lines Deep, and is estimated to cost about €60 per sample. In this case, total cost for transport in our example study would be about €600 000.

Although we do not know any application of the fourth transport plan, home sampling with freezing using one moving collection point which is located close to the participants home so that they can bring their samples would be an option for cohorts recruiting within a restricted geographical region. The cost of this collection effort largely depends on the duration of the sampling scheme and the distance that needs to be covered. When we estimate the cost of a van, dry ice and driver/collector at about €500 a day, sampling a 60-point collection network with 5-day stays would take more than a year and cost about €150 000. With large home–collection point distances, reimbursement of transport costs might need to be implemented, but we did not include this into our estimation. With some additional scheduling efforts, such a transport plan could be combined with medical examination on location. However, short sample collection stays and large home–collection point distances require a lot of flexibility from the study participant and might thus increase drop-out.

Lastly, home sampling with freezing using many collection points together with a courier service has been applied by our lab for the FGFP. In short, we made use of a permanent collection network of 60 local pharmacies equipped with a FGFP freezer, where study participants could deliver their frozen samples during a specified time frame. During the collection period, samples were transported twice a week on dry ice (–70°C) from the collection point to the laboratory, where they were stored at –80°C until further analysis. Initial installation costs of such a network are about €9000, while maintenance takes about €3000. Pharmacies are compensated for their role in the logistic chain with a fee of €2 per sample. One of the largest expenses is the collection point—laboratory transport, which costs about €6500 per collection round covering the complete network. Implementing such a protocol from scratch for 10 000 different samples would thus cost between €58 000 and €136 000 depending on the time frame of the sampling period (2 weeks versus 2 months).

With these data, it is clear that time and cost-efficiency of a complete pre-processing pipeline—including collection, transport and aliquoting—are at this moment no longer determined by whether or not cryopreservation is applied.

While RT protocols applying swabs would in theory be able to collect, transport and aliquot 10 000 samples for as little as ± €35 000 using regular mail transport, stabilizing these samples using an RTTV such as OmniGeneGut would raise the price of such a study to ± €207 000. In comparison, a pre-processing protocol implementing DiviMat together with a transport plan based on a collection network, would cost between €138 000 and €198 000 depending on the collection period (2 weeks versus 2 months) (Box 1). Although transport generally takes the lion share of total pre-processing costs, all three steps (collection, transport or aliquoting) can be the main determinant of cost-efficiency in this stage; it all depends on the applied combination. In contrast, time efficiency of a complete pre-processing pipeline is mainly determined by whether stool samples are divided into aliquots at RT or not, as cutting or drilling a frozen sample takes almost a 10-fold of the time (Table 3).

## FUTURE PERSPECTIVES

Given the small effect size of biologically relevant parameters on gut microbiota composition, discovery research in the gut microbiome field is bound to use collection and preservation methods rendering samples that reflect the original microbiota composition as closely as possible. At this moment, highest sample quality for omics techniques assessing the faecal microbiota can only be obtained with cryopreservation. Future microbiome research would thus largely benefit from improvements in cold-chain management and cost- and time efficiency of protocols implementing this gold standard. This would make it possible to obtain the large number of high-quality samples required for discovery research faster and with fewer resources. Yet, even with advancements in freezing protocols, cryopreservation might not be possible in certain circumstances. The development of RT preservation methods that induce less deviation from the original microbiota composition and/or would allow additional omics techniques would therefore also enhance the field. Especially in the light of targeted studies or follow-up work, such methods might then provide a decent alternative to cryopreservation. However, clear reporting of method comparison studies, with the inclusion of effect size estimations whenever possible, is key in order to make quality assessments and determine whether a method would be suitable to address the research question. Yet, method comparisons are still underreported and effect size information is almost completely unavailable. Time will hopefully change this and enable us to take more informed decisions on sample preservation methods. Increased availability of the necessary information for quality assessment together with improvements in sample pre-processing would certainly facilitate the set up and handling of large-scale gut microbiome studies and would beneficially affect the consistency of their results. Initiatives tackling either of these points would therefore be of great interest to the gut microbiome field and beyond.

## SUPPLEMENTARY DATA

Supplementary data are available at *FEMSRE* online.

fux027_Supp Supplementary data are available at *FEMSRE* online.
