# Supplementary material for: Practical considerations for large-scale gut microbiome studies
Source: FEMS Microbiol Rev. 2017 Jun 30;41(Suppl 1):S154–67. doi: 10.1093/femsre/fux027 (PMC7207147; doi:10.1093/femsre/fux027)
Supplement: fux027_Supp — Supplementary data are available at FEMSRE online. [file fux027_supp.docx]

**METHODS**

**FGFP sampling procedure**

The Flemish Gut Flora Project (FGFP), a large-scale (N>5000) microbiome research project based in Flanders (Belgium), developed a home sampling, aliquotting, and freezing protocol that, in combination with a cold chain collection network, would generate high-quality samples for microbiome research, while at the same time reducing logistic and post-collection analysis expenses.

Participants are invited to enrol through the FGFP project website (<http://www.vib.be/darmflora>) where they can create a password protected FGFP account. This procedure includes an informal consent based on a short description of the study protocol, registration and agreement with the study privacy statement. Next, participants are invited by e-mail to complete a first online questionnaire, collecting data concerning their background (including relations with other volunteers), behaviour, and significant events that could impact the composition and activity of their microbiota (about 45 minutes).

At the start of sample collection periods, sampling packages containing a sampling kit for faecal material and saliva, a medical questionnaire and informed consent are sent to the participants' home addresses by regular mail delivery. Participants schedule an appointment with their general practitioner (GP) who completes the medical questionnaire and takes a blood sample, which is immediately shipped to the contracted blood analysis facility.

After completing the GP’s visit, participants are invited to take faecal samples following the instructions specified in the sampling manual. In short, people are instructed to collect their sample using a biodegradable plastic cover (included in the sampling package), and a self-provided basket and asked to fill three small plastic transparent tubes with about 1cm of stool using the three plastic spoons provided. They could cut off the plastic spoons with a scissor (not provided) and leave the rest in the tube, so that they wouldn’t be left with stool-covered waste. Using the biodegradable plastic they could flush the rest of their stool through the toilet. Participants store the faecal sample vials in a non-transparent plastic bag in their home freezer (-18°C) and deliver their frozen samples, a single copy of the signed informed consent, and the complete medical questionnaire in a collection point of their choice. Therefore 75 local pharmacies were equipped with a FGFP freezer, assuring that every volunteer has access to a recognisable collection point located maximum 10 km of each participant's residence. Twice a week, samples are transported on dry ice (-70°C) from the collection point to the laboratory. Upon arrival, samples are stored at -80°C until further analysis. Shortly after taking the faecal samples, participants are asked to complete a second online questionnaire (about one hour), which aims at taking a snapshot of participants' health, wellbeing, and diet during the weeks preceding sampling.

**Evaluation FGFP sampling procedure**

500 randomly selected FGFP-participants were sent an evaluation questionnaire comprising questions on all aspects of the FGFP-procedure.

**Evaluation of cold-chain management**

A subset of the FGFP sampling kits included a recording device registering sample temperature every 5 minutes. Data of in total 137 temperature recording devices were screened using custom R scripts to determine time lag between home freezing and arrival at the research facility and occurrence of defrosting events during transport.

**Calculations**

**Total time and expenses for the aliquotting of 10.000 samples**

- **Aliquotting after freezing**

±50 samples/day/technician

=> ±200 working days for 10.000 samples

±6€ processing cost/sample (processing cost: bruto wage of €40/hour, 7.5 hours/working day, 50 samples/day/technician = 40 x 7.5 / 50 = €6/sample)

±1€ material cost/sample (cutting or drilling)

=> ± 70.000€

- **Aliquotting before freezing**

±450 samples/day/technician (1 sample/1 min, working-day of 7.5 hours)

=> ±22 working days for 10.000 samples

±0.66€ processing cost/sample (processing cost: bruto wage of €40/hour, 7.5 hours/working day, 225 samples/day/technician = 40 x 7.5 / 225 = 0.66€/sample)

=> ±7000 €
